# System-Level Model and Simulation of a Frequency-Tunable Vibration Energy Harvester

**DOI:** 10.3390/mi11010091

**Published:** 2020-01-14

**Authors:** Sofiane Bouhedma, Yongchen Rao, Arwed Schütz, Chengdong Yuan, Siyang Hu, Fred Lange, Tamara Bechtold, Dennis Hohlfeld

**Affiliations:** 1 Institute for Electronic Appliances and Circuits, Faculty of Computer Science and Electrical Engineering, University of Rostock, Albert-Einstein-Str. 2, 18059 Rostock, Germany; yongchen.rao@uni-rostock.de (Y.R.); chengdong.yuan@jade-hs.de (C.Y.); siyang.hu@jade-hs.de (S.H.); fred.lange@uni-rostock.de (F.L.); tamara.bechtold@jade-hs.de (T.B.); dennis.hohlfeld@uni-rostock.de (D.H.); 2 Department of Engineering, Jade University of Applied Sciences, Friedrich-Paffrath-Str. 101, 26389 Wilhelmshaven, Germany; arwed.schuetz@jade-hs.de

**Keywords:** piezoelectricity, vibration-based energy harvesting, multimodal structures, frequency tuning, nonlinear resonator, bistability, magnetostatic force

## Abstract

In this paper, we present a macroscale multiresonant vibration-based energy harvester. The device features frequency tunability through magnetostatic actuation on the resonator. The magnetic tuning scheme uses external magnets on linear stages. The system-level model demonstrates autonomous adaptation of resonance frequency to the dominant ambient frequencies. The harvester is designed such that its two fundamental modes appear in the range of (50,100) Hz which is a typical frequency range for vibrations found in industrial applications. The dual-frequency characteristics of the proposed design together with the frequency agility result in an increased operative harvesting frequency range. In order to allow a time-efficient simulation of the model, a reduced order model has been derived from a finite element model. A tuning control algorithm based on maximum-voltage tracking has been implemented in the model. The device was characterized experimentally to deliver a power output of 500 µW at an excitation level of 0.5 g at the respected frequencies of 63.3 and 76.4 Hz. In a design optimization effort, an improved geometry has been derived. It yields more close resonance frequencies and optimized performance.

## 1. Introduction

The term energy harvesting is the process of capturing or harvesting wasted or unused ambient energy, such as temperature gradients, mechanical vibrations, radio frequencies, etc., and converting it into useable electrical energy. This differs from powering a system using traditional energy sources such as batteries, fuel cells, etc. Energy harvesting has received much attention in the last two decades from various disciplines, mainly due to its potential impact as a key technology enabling autonomous ultra-low-power electronics operating in remote and harsh environments without the need for large energy storage elements. Energy harvesting thus replaces conventional batteries, enhances the environmental friendliness of the system, and lowers the maintenance costs. In other words, it is a promising technology to power various applications ranging from structural health monitoring to medical implants.

Energy harvesting from mechanical sources including industrial machines, human activity, vehicles, building structures, and other environmental sources can be achieved through different conversion methods. Such approaches include piezoelectric, electromagnetic, electrostatic, and magnetostrictive effects. Among the most promising sources for recovering energy are periodic vibrations generated by rotating machinery or environmental sources such as wind or ocean waves.

The present work addresses vibration-based energy harvesting systems based on the piezoelectric effect. Up to now, the piezoelectric effect has been extensively investigated and integrated in different applications, such as sensors and actuators. Vibration energy harvesting converts vibration energy into electrical energy. The large availability of vibrations in industrial environments and the simultaneous need for sensing applications in such locations motivated the research in recent time.

Most often, a mechanical resonator is used to amplify the low vibration levels into usable deflections. Such systems consist usually of a tip-loaded clamped-free cantilever [1,2,3], which we refer to as ‘first generation harvester concepts’. The drawback of such harvesters is that they operate efficiently only if the harvester’s resonance frequency coincides with the dominant ambient vibration frequency. Any difference between these frequencies will lead to a decrease in the power output. As realistic ambient vibration spectra exhibit multiple frequencies and vary over time as the vibration source is aging or changing in temperature for instance, ‘first generation’ energy harvesting schemes fail. In order to overcome this limitation, many research groups are addressing new resonator designs with an optimized active bandwidth, which enables the harvester to collect power on a broader frequency range. This research can be categorized in two groups. On one hand, numerous multimodal resonator designs, which are able to operate resonantly at multiple frequencies, have been introduced in [4,5,6,7]. Wu et al. [8] investigated a compact piezoelectric energy harvester, comprising of one main cantilever beam and an inner secondary cantilever beam. The system harvests power at two distinct frequencies. A novel trident (three-pronged spear) shaped piezoelectric energy harvester has been proposed by Upadrashta and Yang in [9] to collect power from wideband, low frequency, and low amplitude ambient vibrations. Lamprecht et al. [10] investigated how multiple vibration harvesters can be combined to a macroscopic array configuration by aiming not on high resonance peak powers, but on close spectral overlaps in lower power regions with a bandwidth 500 Hz. In [11] a multiresonant structure comprising a clamped–clamped piezoelectric fiber composite generator has been proposed by Qi et al., with side mounted cantilevers, which are tuned by added masses to resonate at individual frequencies, resulting in a wider harvesting bandwidth. Other works [12,13,14] proposed multiple concepts of multiresonant piezoelectric energy-harvesting devices, capable of harvesting power on a wider frequency range using both translational and rotational degrees of freedom.

On the other hand, other groups proved that integrating nonlinearity in the harvesting device broadens the operative bandwidth compared to the standard linear harvesters. The authors of [15,16,17,18,19,20,21] demonstrated the usability of bistable resonators for harvesting over a wider operational frequency range, by integrating permanent magnets positioned with respect to another permanent magnet on the resonator. In [22] a compact nonlinear multistable energy harvester array has been presented by Lai et al., for harvesting energy at low frequencies. Hoffmann et al. [23,24] developed a harvesting system capable to autonomously adapt the magnetic field strength, acting on the resonator, by adjusting the orientation of a diametrically polarized permanent magnet. Another bistable rotational energy harvester operating at low frequencies has been proposed by Fu and Yeatman in [25,26]. The harvesting is achieved by magnetic plucking of a piezoelectric cantilever using a driving magnet mounted on a rotating platform. In [27,28,29] an autonomous tuning mechanism has been developed, allowing a compensation of the hysteresis, as well as maintaining the optimal working point. It allows the use of both coupling modes (attractive and repulsive), which enables the harvester to adapt its operating frequency to the dominant vibration frequency of the environment. Nammari et al. [30] proposed an enhanced novel design of a nonlinear magnetic levitation-based energy harvester, where the tuning effect is achieved by the magnetic and the oblique springs. In [31,32,33,34] we studied a dual-frequency piezoelectric energy harvester incorporating permanent magnets for bidirectional frequency tuning and presenting an increased useable frequency range compared to standard harvesters. Other groups used different bandwidth broadening strategies, for example, shunted piezoelectric control systems have been proposed in [35,36]. Zhang and Afzalul [37] analyzed a broadband energy harvester with an array of piezoelectric bimorphs mechanically connected through springs. The operative bandwidth broadening can be achieved by carefully selecting the masses and adjusting the spring stiffness.

In the present paper, we propose a so-called ‘folded beam’ resonator design for energy harvesting. It consists of an outer tip-loaded beam, mechanically connected to a pair of inner beams, which extend towards the fixed end. In contrast of [8], the resonator is fabricated from a single steel sheet. It uses permanent magnets at the free ends of the cantilevers for higher frequency agility, by making use of the nonlinearity provided for the nonlinear magnetic forces. It resonates at two distinct frequencies, spaced 10 Hz apart, in the range (50,100) Hz. Bidirectional frequency tuning was achieved using a similar approach as presented in [34]. It has been demonstrated that both modes can be tuned independently, together with the dual frequency feature, can provide a superior frequency agility and increase the operational bandwidth of the system compared to existing approaches. Furthermore, an optimized version of the resonator is presented in this paper, where the two power peaks could be nearer to each other and provide the same power levels. This feature further enhances the performances of such a harvester and shows the benefit of such a design compared to an array of simple harvesters. Due to the close resonances enabling the resonator to amplify the deformation of the outer beam and leading to a higher strain distribution on the inner beam compared to the aforementioned simple design, where the second cantilever will be directly connected to the same excitation. A control scheme, which uses an energy efficient maximum- amplitude tracking algorithm, is optimized compared to [34]. A reduced model of the harvester has been developed and enabled us to fully simulate the behavior of the harvester in question. The position-dependent magnetic force obtained from magnetostatic simulations presented in [33] have been implemented together with the reduced model in ANSYS twin builder. The system is able to autonomously choose and tune the closest mode to the dominant vibration frequency to maintain maximum possible oscillation amplitude. Furthermore, we dropped the self-adaptive step size tuning control scheme due to the potential of higher power requirements needed for the fine-tuning operation. Finally, we experimentally characterized the behavior of the first prototype of such a piezoelectric harvester design and the result matched with the simulation results in terms of frequency and expected voltage output.

## 2. Dual Frequency Piezoelectric Energy Harvester

This section reviews shortly the dual-frequency harvester proposed in [32,33,34] together with its tuning approach. Continuing our modelling effort from [34] we advance the finite element (FE) model into a system-level model. For this purpose, we derive a reduced order model, integrate nonlinear tuning and damping forces, a control algorithm, and electrical circuitry into a more complex model.

### 2.1. Design Description

**Figure 1:**
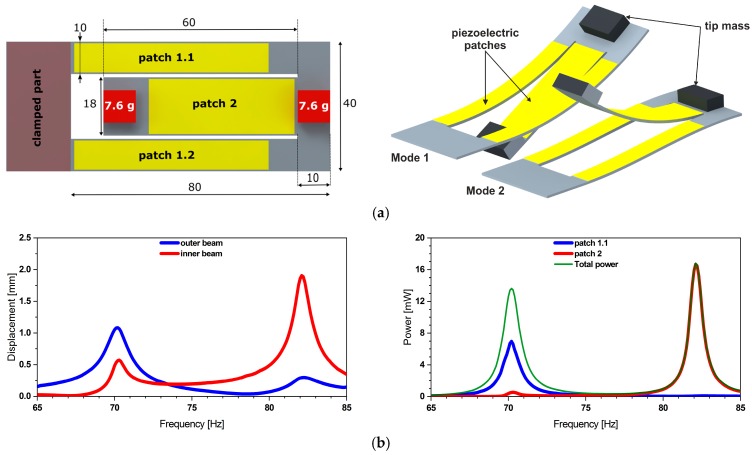
(**a**) Geometry description of the harvester design together with (**b**) its simulated displacement, respectively power output. The transfer function illustrates the dual frequency operation of the structure under a base acceleration of 0.5 g. Simultaneously, it demonstrates comparable power output levels.

The mechanical resonator design consists of two identical 80 mm long arms (referred to as outer beams), mechanically coupled through a common end to a 60 mm long inner beam, which extends in turn towards the fixed end [34]. The tip masses are of identical weight *m* = 7.6 g. The first two resonance frequencies and mode shapes, as obtained from a harmonic analysis, are shown in Figure 1. The deformation of the piezoelectric layers results in a surface charge distribution and consequently a voltage across the patch electrodes. The maximum power output of the harvester can be derived from this voltage and the patch capacitance as explained in [34]. The phase difference between the inner and outer patch depends on the frequency of operation. We identified the phase difference to be 200 and 30° at the first, respectively the second mode. A connection of both patches shall consider and adapt the polarity of the voltage output. Realistic vibration excitation might excite both modes simultaneously so that individual power processing is mandatory.

In order to increase the frequency agility of the system, we propose magnetic frequency tuning. The approach uses permanent magnets on the resonating structure together with fixed external magnets. The interaction between the fixed and movable magnets creates an attracting or repelling force, which adds to the mechanical restoring force of the structure. The change in stiffness of the structure leads to a frequency up- or down-tuning. In [34] we characterized the resonator and demonstrated bidirectional frequency tuning of 15%. In order to simulate the effect of the magnetic force, the use of the full FE model is not efficient anymore, due to the very long solution time needed for such a simulation. Therefore, in [34] we proposed a compact model of the resonator and we were able to fully simulate the behavior of such a system. However, the integration of the piezoelectric transducer elements is not feasible with a lumped modelling approach. Therefore, in this paper, we derive a reduced order model of the full FE model and implement it in a system-level simulation.

### 2.2. Reduced Order Model of the Piezoelectric Energy Harvester

The piezoelectric energy harvester model implemented in ANSYS^®^ Mechanical (V2019, R1) was thoroughly described in [34]. Considering its high computational cost for a transient simulation, Krylov subspace-based model order reduction (MOR) methods, also known as rational interpolation [38,39,40] were implemented to generate a highly compact but accurate reduced order model (ROM). Furthermore, based on this ROM, a circuit-device co-simulation of the piezoelectric energy harvester became feasible in the system-level simulation.

#### Model Order Reduction

The finite element method provides a spatially discretized mathematical description of the piezoelectric energy harvester. It is represented by a second order multiple-input multiple-output (MIMO) system of the form:

(1) $$\sum_{N}\{\begin{array}{c} \underset{M}{\underbrace{\left[\begin{array}{cc} M_{11} & 0\\ 0 & 0 \end{array}\right]}}\left[\begin{array}{c} {\ddot{x}}_{1}\\ {\ddot{x}}_{2} \end{array}\right]+\underset{E}{\underbrace{\left[\begin{array}{cc} E_{11} & 0\\ 0 & 0 \end{array}\right]}}\left[\begin{array}{c} {\dot{x}}_{1}\\ {\dot{x}}_{2} \end{array}\right]+\underset{K}{\underbrace{\left[\begin{array}{cc} K_{11} & K_{12}\\ K_{21} & K_{22} \end{array}\right]}}\underset{x}{\underbrace{\left[\begin{array}{c} x_{1}\\ x_{2} \end{array}\right]}}=\underset{B}{\underbrace{\left[\begin{array}{c} B_{1}\\ B_{2} \end{array}\right]}}u\\ y=\underset{C}{\underbrace{\left[\begin{array}{cc} C_{1} & C_{2} \end{array}\right]}}\left[\begin{array}{c} x_{1}\\ x_{2} \end{array}\right] \end{array}$$

where $M,E,K\in {\mathbb{R}}^{N\times N}$ are the mass, damping, and stiffness matrices, respectively. $B\in {\mathbb{R}}^{N\times p}$ and $C\in {\mathbb{R}}^{q\times N}$ are the input and gathering matrices, with $u\in {\mathbb{R}}^{p}$ and $y\in {\mathbb{C}}^{q}$ being user defined input and output vectors. $x_{1}$ and $x_{2}$ present the nodal displacement and electrical potentials in the state vector $x\in {\mathbb{C}}^{N}$. Considering the large size of system (1) obtained in this work (N = 166,789 DoF), a highly compact but accurate ROM was generated:

(2) $$\sum_{r}\{\begin{array}{c} \underset{M_{r}}{\underbrace{V^{T}MV}}\cdot \ddot{z}+\underset{E_{r}}{\underbrace{V^{T}EV}}\cdot \dot{z}+\underset{K_{r}}{\underbrace{V^{T}KV}}\cdot z=\underset{B_{r}}{\underbrace{V^{T}B}}\cdot u\\ y=\underset{C_{r}}{\underbrace{CV}}\cdot z \end{array}$$

where $V\in {\mathbb{R}}^{N\times r}$ is the orthonormal basis of the second order Krylov subspace $K_{r}\left(-K^{-1}E,-K^{-1}M,-K^{-1}B\right)$ obtained through the Second Order Arnoldi Reduction (SOAR) method [41,42]. The full-scale state vector x is approximated by x≈V·z, where $z\in {\mathbb{C}}^{r}$ is the reduced state vector,r=35≪N.

In the previous research [43,44,45], the stability of the reduced system (2) could not be guaranteed by the conventional MOR methods. Several stabilization approaches have been introduced and mathematically proven in [46,47,48]. In this work, we apply the efficient approach ’Schur after MOR’ to generate a stable ROM of the piezoelectric energy harvester model. “Schur after MOR” projects the reduced system (2) onto a sorted orthonormal eigenbasis of matrix $M_{r}$:

(3) $${\tilde{\sum }}_{r}\{\begin{array}{c} \underset{{\tilde{M}}_{r}}{\underbrace{T^{T}M_{r}T}}\cdot \ddot{q}+\underset{{\tilde{E}}_{r}}{\underbrace{T^{T}E_{r}T}}\cdot \dot{q}+\underset{{\tilde{K}}_{r}}{\underbrace{T^{T}K_{r}T}}\cdot q=\underset{{\tilde{B}}_{r}}{\underbrace{T^{T}B_{r}}}\cdot u\\ y=\underset{{\tilde{C}}_{r}}{\underbrace{C_{r}T}}\cdot q \end{array}$$

where $T\in {\mathbb{R}}^{r\times r}$ and ${\tilde{M}}_{r}$ are the eigenvector matrix and sorted diagonal eigenvalue matrix of $M_{r}$, obtained through eigen decomposition of $M_{r}=T{\tilde{M}}_{r}T^{T}$. According to the indexes of the relative small eigenvalues in ${\tilde{M}}_{r}$, all the system matrices in (3) can be partitioned:

(4) $$\begin{array}{l} {\tilde{M}}_{r}=\left[\begin{array}{cc} {\tilde{M}}_{1} & 0\\ 0 & {\tilde{M}}_{2} \end{array}\right]\approx \left[\begin{array}{cc} {\tilde{M}}_{1} & 0\\ 0 & 0 \end{array}\right];\;{\tilde{E}}_{r}=\left[\begin{array}{cc} {\tilde{E}}_{11} & {\tilde{E}}_{12}\\ {\tilde{E}}_{21} & {\tilde{E}}_{22} \end{array}\right]\approx \left[\begin{array}{cc} {\tilde{E}}_{11} & 0\\ 0 & 0 \end{array}\right];\\ {\tilde{K}}_{r}=\left[\begin{array}{cc} {\tilde{K}}_{11} & {\tilde{K}}_{12}\\ {\tilde{K}}_{21} & {\tilde{K}}_{22} \end{array}\right];\;{\tilde{B}}_{r}=\left[\begin{array}{c} {\tilde{B}}_{1}\\ {\tilde{B}}_{2} \end{array}\right];\;{\tilde{C}}_{r}=\left[\begin{array}{cc} {\tilde{C}}_{1} & {\tilde{C}}_{2} \end{array}\right] \end{array}$$

where ${\tilde{M}}_{1},{\tilde{E}}_{11},{\tilde{K}}_{11}\in {\mathbb{R}}^{I\times I}$, ${\tilde{M}}_{2},{\tilde{E}}_{22},{\tilde{K}}_{22}\in {\mathbb{R}}^{\left(r-I\right)\times \left(r-I\right)}$, ${\tilde{E}}_{12},{\tilde{K}}_{12}\in {\mathbb{R}}^{I\times \left(r-I\right)}$, ${\tilde{E}}_{21},{\tilde{K}}_{21}\in {\mathbb{R}}^{\left(r-I\right)\times I}$, ${\tilde{B}}_{1}\in {\mathbb{R}}^{I\times p}$, ${\tilde{B}}_{2}\in {\mathbb{R}}^{\left(r-I\right)\times p}$, ${\tilde{C}}_{1}\in {\mathbb{R}}^{q\times I}$, ${\tilde{C}}_{2}\in {\mathbb{R}}^{q\times \left(r-I\right)}$, and I∈[1,r]. In order to reconstruct the reduced system in an analogous manner as system (1), the relatively small part ${\tilde{M}}_{2}$ and parts ${\tilde{E}}_{12},{\tilde{E}}_{21},{\tilde{E}}_{22}$ in (4) are all neglected. In this way, the Schur complement transformation can be performed and the stable piezoelectric energy harvester ROM is obtained:

(5) $${\tilde{\sum }}_{r\_Schur}\{\begin{array}{c} {\tilde{M}}_{1}\cdot {\ddot{q}}_{1}+{\tilde{E}}_{11}\cdot {\dot{q}}_{1}+\left({\tilde{K}}_{11}-{\tilde{K}}_{12}{\tilde{K}}_{22}^{-1}{\tilde{K}}_{21}\right)\cdot q_{1}=\left({\tilde{B}}_{1}-{\tilde{K}}_{12}{\tilde{K}}_{22}^{-1}{\tilde{B}}_{2}\right)\cdot u\\ y=\left({\tilde{C}}_{1}-{\tilde{C}}_{2}{\tilde{K}}_{22}^{-1}{\tilde{K}}_{21}\right)\cdot q_{1}+\left({\tilde{C}}_{2}{\tilde{K}}_{22}^{-1}{\tilde{B}}_{2}\right)\cdot u \end{array}$$

### 2.3. System-Level Simulation

**Figure 2:**
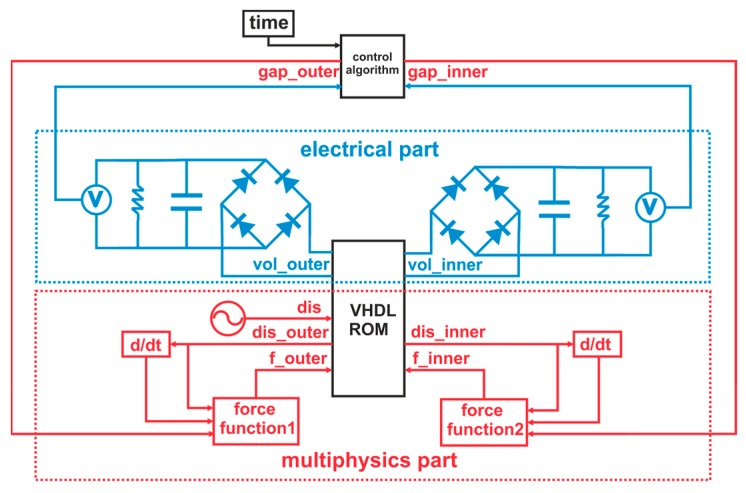
System-level model implemented in ANSYS twin builder, including a reduced order model of the harvester together with tuning actuation, the conditioning circuitry, and the tuning control algorithm.

The system-level model consists of three parts as depicted in Figure 2. The multiphysics part comprises the ROM, which describes the mechanical and piezoelectric behavior of the harvester. The magnetic forces have been derived from magneto-static simulations [33] and implemented in the model as force functions 1 and 2. The magnetic force will alter the effective stiffness of the resonator. The damping ratio of the mechanical resonator depends on the structure’s stiffness. Hence, it also varies while tuning. We address this by adjusting the damping ratio accordingly. The electrical part encompasses the rectification circuitry. A tuning control algorithm, based on maximum amplitude tracking, is included as well.

#### 2.3.1. Mechanical Resonator Reduced Order Model

**Figure 3:**
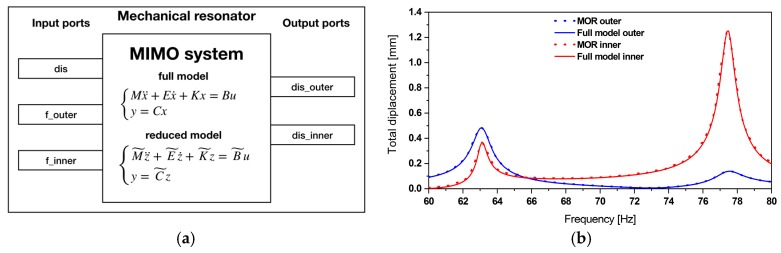
(**a**) The multiple-input multiple-output (MIMO) system of the mechanical resonator together with (**b**) the resonator reduced model validation through a comparison with the full finite element (FE) model. Both models are subjected to an excitation amplitude of 10 μm.

This subsection presents the validation of the reduced order model by comparison between results of a FE model, the reduced order model and experimental data. The MIMO system of the resonator is illustrated in Figure 3. Both models use three inputs: Base excitation “dis”, (displacement amplitude of the ambient vibration), force on outer and inner beam “f_outer” and “f_inner”, respectively. The tip displacement of the outer and inner beam “dis_outer” and “dis_inner” are the two outputs. The displacement amplitudes of the outer and inner beam of the FE model, respectively the reduced order model, are shown in Figure 3 for a displacement amplitude of 10 µm at the clamped part.

The obtained results from the reduced order model matched well with the FE model. The two fundamental modes appear at 63.1 and 77.5 Hz. Furthermore, the aforementioned frequencies have been compared to the experimental results obtained in [33], which are 62.6 and 76.1 Hz.

**Figure 4:**
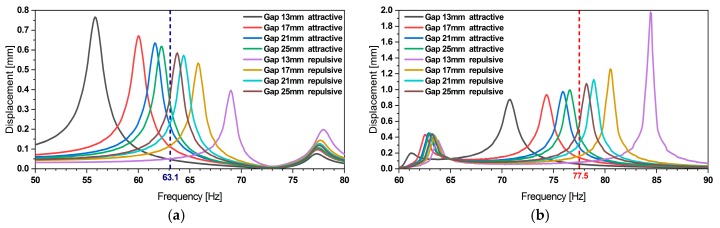
(**a**) Variation of the displacement amplitude of outer and (**b**) inner beam during frequency tuning. An 18% of bidirectional frequency shift can be achieved. The data indicates that frequency tuning does not affect the other resonance frequency.

In [32,34] we presented a description of the magneto-static simulations, which enabled us to derive the magnetic forces involved in the frequency tuning. The same forces have been considered in the current system-level model. As shown in Figure 4 the magnetic forces yield a bidirectional frequency shift by up to 18%.

**Figure 5:**
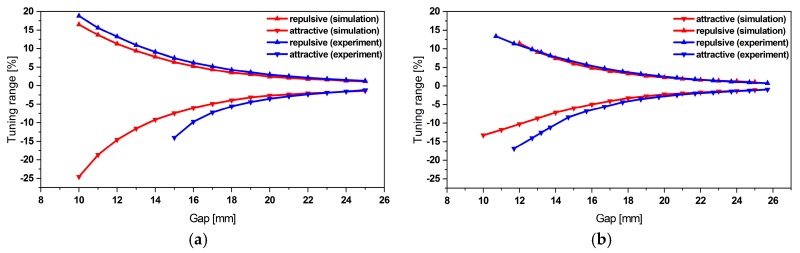
(**a**) Experimental validation of the bidirectional frequency tuning simulation of first and (**b**) second resonance frequency.

The frequency tuning system-level simulations showed a relative error of approximately 3.1% and 1.8% for the first, respectively the second mode tuning (see Figure 5) when compared to experimental data. Yet, tuning towards smaller frequencies reveals an increasing discrepancy. We attribute this to some limitations of the underlying magnetostatic model, which neglects the rotation and lateral displacement of the magnet as the resonator undergoes deflection. Furthermore, as displacement amplitude increases while tuning towards smaller frequencies such deviations show a more pronounced effect.

#### 2.3.2. Piezoelectric Energy Harvester Reduced Order Model

**Figure 6:**
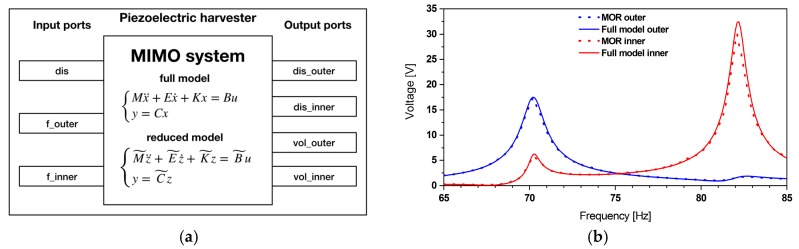
(**a**) The MIMO system of the piezoelectric energy harvester and (**b**) the harmonic response of the reduced order and full FE model subjected to a 10 μm excitation amplitude.

After the validation frequency-agile resonator model, a reduced order model of the piezoelectric energy harvester has been derived. The corresponding MIMO system is illustrated in Figure 6.

Compared to the mechanical resonator model, the piezoelectric energy harvester model has two additional outputs “vol_outer”, “vol_inner”, referring to the voltage levels at the piezoelectric patches on the outer and inner beams. The results obtained from the reduced order and the FE model fit well and thus support the applicability of this reduced order modeling approach.

#### 2.3.3. Electrical Simulation

The system-level model integrates the reduced order model with electrical circuitry. Here, we considered a diode bridge for full-wave rectification, a capacitor for filtering, a buck converter for voltage regulation, and a resistive load with optimum resistance. The circuitry is connected to the two electrical ports of the reduced order model.

##### Rectification and Filtering

**Figure 7:**
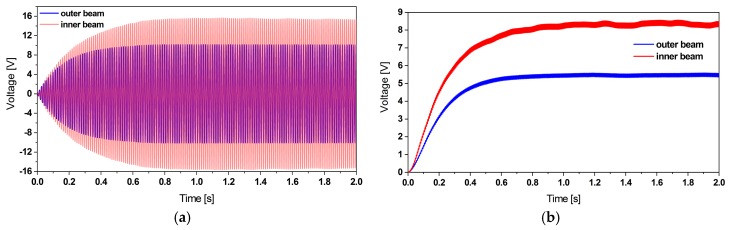
(**a**) Simulation results of the AC voltage output before rectification and (**b**) the filtered DC voltage output of the piezoelectric harvester subjected to 0.2 g base excitation.

As presented in Figure 7, after rectification and filtering the AC voltage output, a DC voltage output with small ripple voltage is obtained.

##### Optimum Load

**Figure 8:**
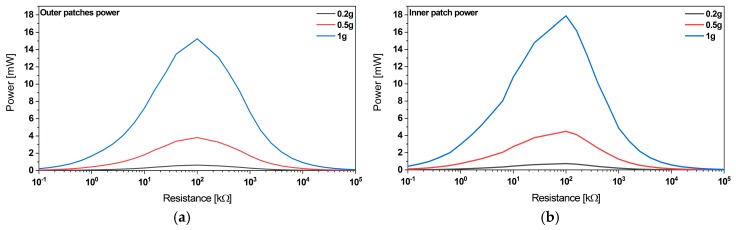
(**a**) Load matching to ensure maximum power output from the outer patches electrically connected in the series, (**b**) respectively the inner patch.

Due to the capacitance of the piezoelectric patches, the power output depends on the load resistance. To ensure maximum power delivery, the optimum load has been found to be 100 and 60 kΩ for the outer and inner beam, respectively (see Figure 8). The power in the load is proportional to the square of the excitation amplitude. Therefore, we performed the simulations at several excitation levels.

##### Voltage Regulation

**Figure 9:**
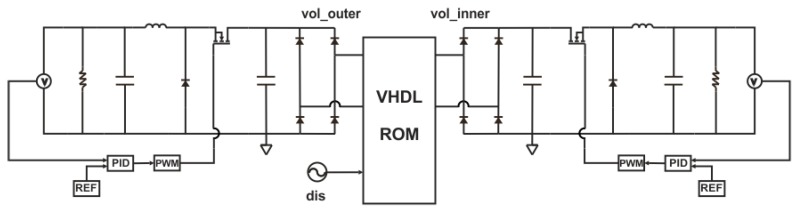
Two independent buck converter circuits for voltage regulation.

Piezoelectric energy harvesters, which employ PZT as a piezoelectric material, easily generate voltage levels, which exceed the range compatible with electronic circuits and related components, such as microcontrollers or super capacitors. Voltage regulation is required for reliable operation. The voltage regulation is achieved, e.g., by using a buck converter, which includes a MOSFET, a capacitor, an inductor, and a diode as shown in Figure 9. The duty cycle of the MOSFET’s state affects the voltage output in such a circuit. A controller alters the duty-cycle to maintain constant output voltage for varying loading situations.

**Figure 10:**
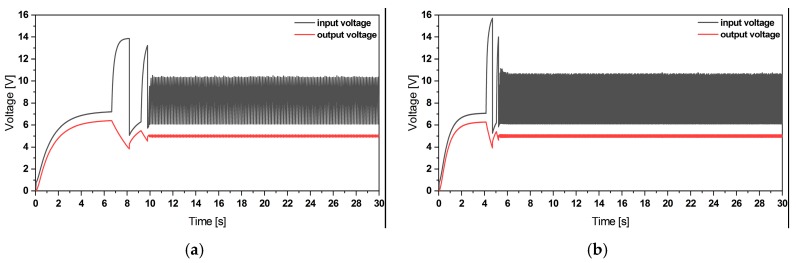
(**a**) Simulation results of the voltage regulation of the outer patches and (**b**) the inner one under 0.2 g base excitation.

Figure 10 shows the simulation results of the voltage regulation. Here, the voltage at the outer and inner beam have been regulated to 5 V within 10 s.

#### 2.3.4. Energy Harvester Frequency Tuning

**Figure 11:**
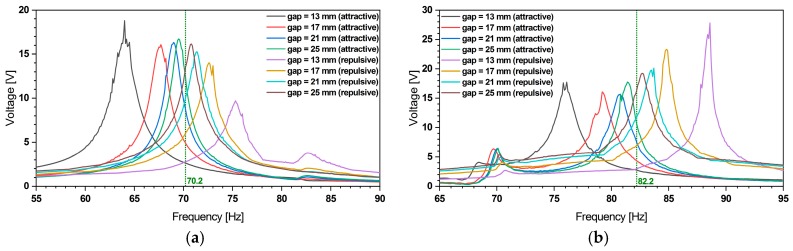
(**a**) The voltage amplitude variation of the piezoelectric energy harvester under bidirectional magnetic frequency tuning of first and (**b**) second resonance frequency at 0.2 g harmonic base excitation.

We also studied the effect of our frequency tuning mechanism on the voltage output in analogy to the procedure described in Section 2.3.1. The results of the system-level simulation are shown in Figure 11.

These results show the voltage amplitude variation as a result of the bidirectional frequency shift of the piezoelectric harvester. These simulations show bidirectional frequency tuning by up to 9% relative to the unaltered resonance frequencies. The reduced tuning range is due to the increased beam stiffness induced by the piezoelectric patches.

#### 2.3.5. Control Algorithm

**Figure 12:**
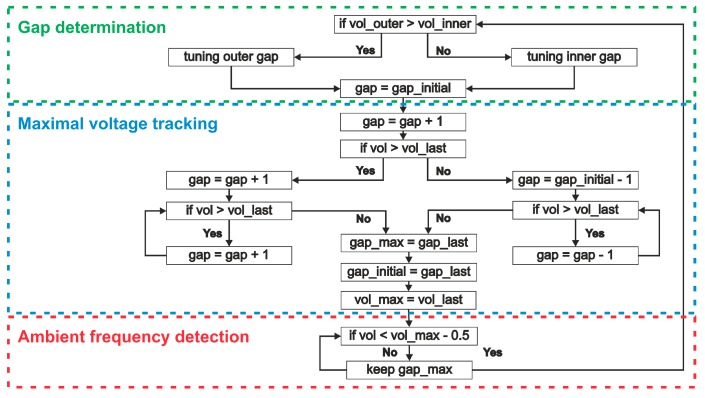
Tuning control algorithm scheme based on maximum-voltage tracking.

In this section, we focus on the tuning control as implemented in the system-level model of Figure 2. A control algorithm, as visualized in Figure 12, maintains maximum voltage output even under varying excitation frequency. We consider this as a realistic use case. The time variable and DC voltage output are used as inputs for the control algorithm. The control scheme analyzes the voltage levels at a given frequency and selects one of the two tuning mechanisms.

**Figure 13:**
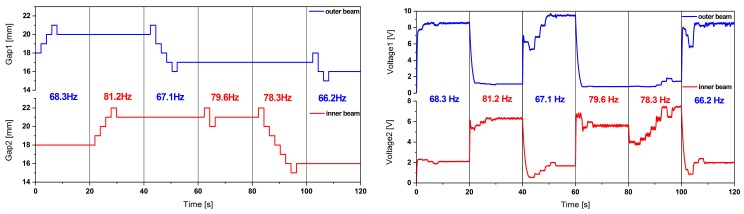
A control algorithm, which is based on maximum-voltage tracking, chooses the most effective tuning actuator.

In order to demonstrate the self-adaption of our harvester, we excite the structure with a stepwise frequency-varying harmonic excitation, as illustrated in Figure 13. The results demonstrate that the system is able to choose the adequate tuning mechanism. This choice is based on the current voltage level. The maximum voltage is achieved within 15 s. In contrast to our previous work [33], where we implemented an adaptive step size, here the tuning scheme employs a constant step size. This results in less tuning steps, which improve the energy efficiency of the tuning mechanism.

## 3. Experimental Investigation

**Figure 14:**
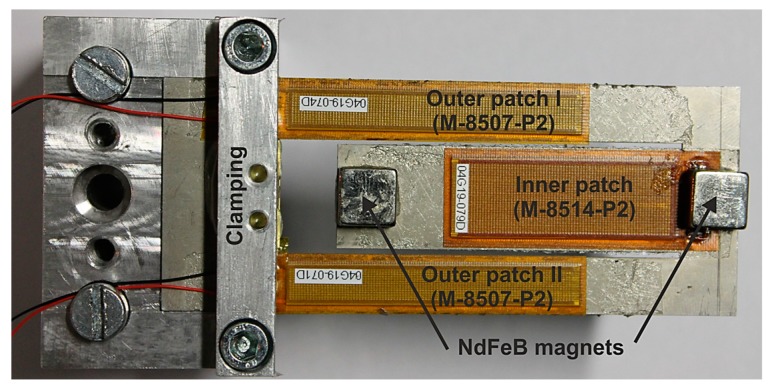
Dual frequency piezoelectric energy harvester with macro fiber composite (MFC) patches on outer and inner beams. Permanent magnets are attached at the beam ends. The base excitation is applied to the clamped part on the left side.

In order to characterize the presented harvester design, we integrate three macro fiber composite (MFC) piezoelectric patches supplied by SMART MATERIAL Corp. (M-8507-P2 on the outer beams and M-8514-P2 on the inner beam) with a resonator fabricated from steel, as depicted in Figure 14. The MFC patches are composed of piezoelectric rods embedded between layers of adhesive, interdigitated electrodes, and encapsulated with polyimide film. The patch dimensions are 60 × 7 × 0.18 mm³ and 48 × 14 × 0.18 mm³ for the outer and inner beam, respectively. The material properties of the MFC patches are given in Table 1.

**Figure 15:**
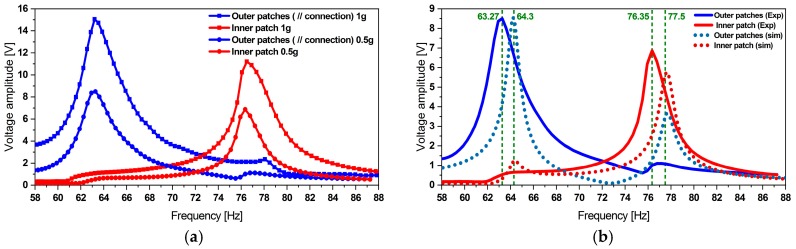
(**a**) Experimentally obtained voltage at the excitation levels of 0.5 and 1.0 g. Comparison of experimental data and (**b**) simulation results for an excitation level of 0.5 g.

The harvester has been excited at the acceleration amplitudes of 0.5 and 1.0 g. The results presented on Figure 15 demonstrate the dual frequency feature of the harvester. The experimentally observed resonance frequencies *f*_1 *Exp*_ = 63.27 and *f*_2 *Exp*_ = 76.35 Hz match the simulation results *f*_1 *Sim*_ = 64.30 and *f*_2 *Sim*_ = 77.50 Hz. However, a lower voltage output has been observed. We attribute this to the adhesive tape which attaches the patches to the steel. In our assembly this degrades the strain transfer between the steel resonator and the piezoelectric layers when compared to solid bonding, e.g., using glue. Our simulations considered the adhesive tape as a material of high compliance (*E* = 450 kPa). The FE model implements a constant damping ratio, which yields correct amplitudes at the first mode and does not describe the damping at the second mode. A mode-specific or even frequency dependent damping ratio shall be applied instead. Furthermore, the slight frequency shift (up to 1.63%) between the model and the experiment results is caused by the additional mass of the solder paste used to electrically connect the inner patch. The patch attachment procedure and the limited reproducibility of the magnets positioning contribute in turn to such a frequency shift.

**Figure 16:**
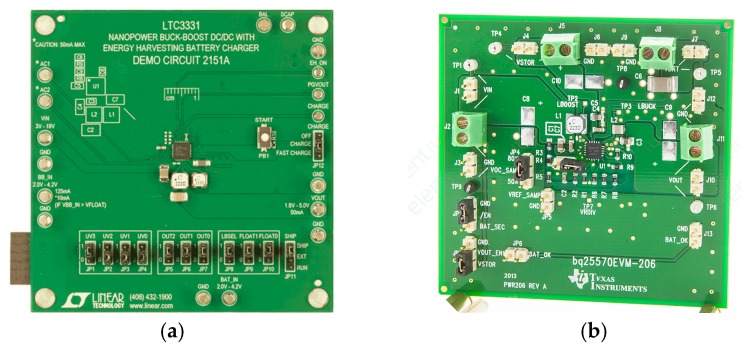
(**a**) Power management boards 2151A from analog devices and (**b**) bq2557OEVM-206 from Texas Instruments used as power management circuits.

We evaluated the harvester’s power delivery using the power management board 2151A provided by analog devices (see Figure 16), which also enables battery charging. The board integrates the LTC3331 chip, which provides a regulated voltage from various energy harvesting sources. The circuitry consists of an integrated low-loss full-wave bridge rectifier and a buck converter. The rechargeable coin-cell battery powers a buck-boost converter capable of providing voltages between 1.8 and 5.0 V. Depending on the available power from the harvester the board is either supplied by the harvester or the battery. An internal prioritizer switches between the power sources. If the harvesting source is available, the buck converter is active and the buck-boost is off and vice versa.

The power management board 2151A has been tested under different configurations as presented in Table 2.

Furthermore, we evaluated the efficiency of different power management boards the 2151A and the bq25570EVM-206, designed for low power applications and providing only 1.8 V output voltage as depicted in Table 3. The efficiency in this case is nothing than the ratio between the output and the input power.

The experiments revealed that a maximum efficiency of approximately 50% can be reached using both boards with our harvester.

## 4. Parametric Design Optimization

One of the key features of the presented folded beam harvester design is the possibility to enhance the overall performance if the first two resonance frequencies appear closely spaced frequencies (co-resonance) and simultaneously provide the same power levels. This is a unique feature not provided by other multiresonant structures such as an array of two beams. All cantilevers of an array are subjected to the same base excitation, whereas in the case of the coupled resonator, the inner beam is subjected to the maximum tip displacement of the outer one, which is higher than the applied base excitation. This motivated us to investigate the possibility of optimizing the existing design.

**Figure 17:**
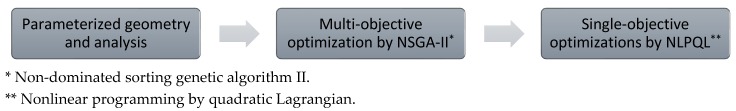
Optimization process.

The geometry of a vibration energy harvester determines its dynamic properties and thereby its operating frequency and the harvested power. Consequently, optimized dimensions yield higher power and better performance. For this purpose, the reference design was parameterized and optimized for an operating bandwidth centered at 75 Hz. The process of this optimization relied on FE models and is shown in Figure 17.

**Figure 18:**
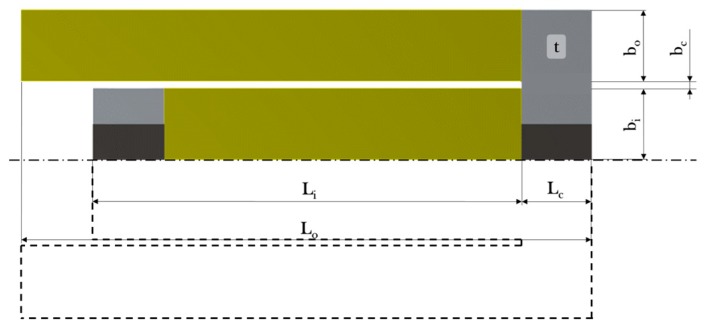
Parameterization of the reference geometry (light grey corresponds to steel, black to NdFeB, and yellow to PIC255).

Firstly, the reference geometry was parameterized and subsequently optimized. Figure 18 presents the parameterized model. Table 4 gives the range of the seven geometry parameters. The size of the magnets and their positions was unchanged during the optimization process. A parameter range of ±50% has been chosen with respect to the reference design. The thickness t is a discrete parameter, because the device is fabricated from a metal sheet, which is available only at certain thickness values. The parameter $L_{i}$ has bounds chosen to enable efficient usage of space for all values of $L_{o}$. Constraining $L_{i}$ prevents the inner beam to overlap with the fixed support.

A modal analysis and a harmonic analysis have to be performed to compute the objective values of the optimization. A modal analysis determines the eigenfrequencies for the first two modes, while a harmonic analysis computes the electrical power at these modes. The harmonic analysis implements a damping ratio of 0.8% which has been determined experimentally for a similar design. The base excitation acceleration amplitude was 0.01 g. The two outer piezoelectric elements were connected in parallel. The inner element was connected in series in order to obtain maximum power at an optimized load resistance. The results of these analyses yield the parameters and objectives presented in Equations (6)–(13) of which Equations (6)–(9) give the vibrational properties:

(6) $$\bar{f}=\frac{f_{1}+f_{2}}{2}$$

(7) $$obj\;f=\left|75\;Hz-\bar{f}\right|$$

(8) $$\Delta f_{rel}=\frac{f_{1}-f_{2}}{\bar{f}}$$

(9) $$obj\;\Delta f_{rel}=\left|0.05-\Delta f_{rel}\right|.$$

Here, $f_{1}$ and $f_{2}$ are the first two eigenfrequencies, $\bar{f}$ is their mean value and $\Delta f_{rel}$ is the relative operating frequency range. The two objectives obj f and $\Delta f_{rel}$ describe the intended operating frequency range. Upper bounds of 0.05 for $obj\;\Delta f_{rel}$ and 5 Hz for obj f control the convergence of the optimization algorithm.

Equations (10)–(13) are related to the electrical behavior:

(10) $$V=-g_{31}\;t\;\sigma_{1}$$

(11) $$P=2\pi V^{2}\frac{C_{o}C_{i}}{2C_{o}+C_{i}}f$$

(12) $$obj\;P\;ratio=\frac{\min\left(PD_{1},PD_{2}\right)}{\max\left(PD_{1},PD_{2}\right)}$$

(13) $$obj\;\bar{PD}=\frac{PD_{1}+PD_{2}}{2},$$

where V is the approximated voltage of a piezoelectric patch, $g_{31}$ is the piezoelectric voltage coefficient for the 31 mode, t is the thickness of the piezoelectric patch, $\sigma_{1}$ is the normal stress due to bending, P is the electrical power in an attached resistor of at optimum load value, $C_{o}$ and $C_{i}$ are the capacitances of the inner and outer piezoelectric patches, f is the frequency and $PD_{1}$ and $PD_{2}$ are the power densities at the first and second eigenfrequency, respectively. The voltage was obtained analytically from the mechanical model in order to reduce the computational effort. This neglects the electromechanical back coupling. The power objectives obj P ratio and $obj\;\bar{PD}$ evaluate the frequency spacing of the two maxima and their amplitude ratio.

The optimization follows a two-step procedure: A global multi-objective optimization and subsequent local single-objective optimizations. Methods to decrease the computational effort such as a sensitivity analysis or a metamodel were omitted, as they were suffering from insufficient accuracy. The large design space and the nonlinear objective space require this two-step procedure where the first step searches for promising subspaces. A second, more refined step searches this subspace to find the final candidates. The multi-objective optimization employs the evolutionary algorithm NSGA-II, which iteratively evolves a set of start designs by selection, crossover, and mutation to satisfy the objectives of the optimization. The start population contained 3500 designs; each following generation comprised 100 designs. A crossover probability of 98% and a mutation probability of 1% defined the reproduction. The optimization converged for either 20 generations, a convergence stability of 2% or if 70% of the designs of a generation were Pareto-optimal. The Pareto set provides one start design per thickness for the single-objective optimizations. These start designs were selected to satisfy the two vibrational objectives to guarantee operation at resonance and broaden the harvesting. A subsequent single-objective optimization relied on the algorithm NLPQL for a gradient-based optimization. The local search deployed central differences and a finite difference of 1%. The parameter ranges for each single-objective optimization were ± 10% of the start design. These local optimizations changed the definition of $obj\;\Delta f_{rel}$ to $obj\;\Delta f_{rel}=\left|0.01-\Delta f_{rel}\right|$. The optimization was considered completed if the change for the next iteration fell below 0.1% or if 20 iterations were reached. The local optimization comprised up to three single-objective optimizations.

**Figure 19:**
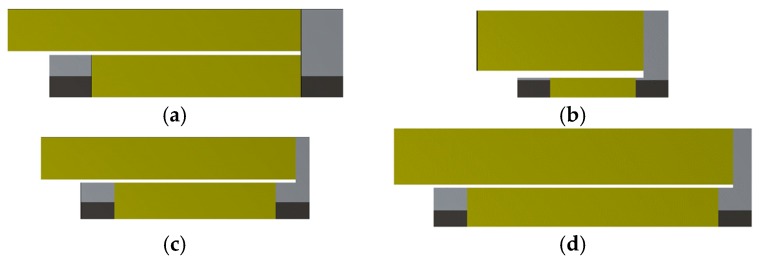
(**a**) Reference geometry and (**b**) the three optimized designs with thicknesses of t=0.5, (**c**) t=1, and (**d**) t=1.5 mm.

Figure 19 presents the geometry of the reference design together with the individual optimized designs of all three thicknesses. The optimized design (*c*) can be compared to the reference design since both have the same thickness. Important differences are the length of the connection and the width of the outer beam, which results in an operating frequency close to 75 Hz.

**Figure 20:**
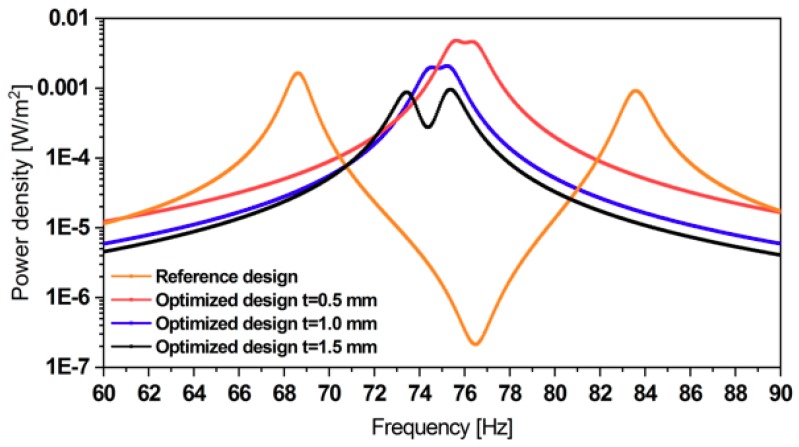
Power density of the reference design and the optimized designs. The co-resonance results in an extended operative bandwidth at comparable power levels.

Figure 20 compares the power densities of the four designs in Figure 19. The power density of the reference design has two dominant peaks. However, the power drops beyond the bandwidth. In contrast, the designs (a) and (b) provide an operational frequency range with a power variation of only 3.5% centered at 75 Hz. Moreover, those designs also have higher peak power densities since their more thin steel structure is more compliant. Up to three local optimizations were performed for each design. Hence, a higher number of local optimizations will further improve the designs.

In summary, an optimized geometry provides equal power at both resonance frequencies at even higher power density, as demonstrated with the 0.5 mm thick design.

## 5. Conclusions

This work advances the research work presented in [33]. We present a novel self-tunable dual-frequency piezoelectric energy harvester with optimized performances. The dual frequency feature has been thoroughly investigated and we demonstrated that the resonator magnifies the amplitudes at two close fundamental frequencies, enabling simultaneous energy harvesting from both vibration frequencies. The system integrates permanent magnets, whose magnetostatic forces enable the frequency agility of the harvester. In order to simulate the bidirectional frequency tuning effect, we derived a reduced order model of the resonator and the harvester finite element model. The resonator’s reduced order model has been experimentally validated and we demonstrated ±18% of bidirectional tuning. Furthermore, the reduced order modelling has been applied to the harvester.

A control algorithm has been developed to drive the tuning mechanism and thereby ensures the self-adaption of the system. The algorithm is based on maximum-voltage tracking and is able to automatically choose the adequate tuning actuator. 

Furthermore, we presented experimental results of the piezoelectric harvester. The characterization demonstrated the dual-frequency feature of the harvester and showed that the harvester supplies sufficient voltage and power levels. Additionally, we investigated the efficiency of two commercially available power management systems. Further experiments will be performed to evaluate the harvesting system’s performance under realistic applications.

Finally, an optimized version of the harvester design has been proposed. This design presents two modes appearing at two close frequencies and an increased operative bandwidth. Further experiments are planned to verify the characteristics of the optimized version.

## Figures and Tables

**Table 1:** Material properties of the active area of the macro fiber composite (MFC) patches.

Material Properties	Value
Mass density (kg/m^3)^	5440
Tensile modulus, *E*_1_ (rod direction) (GPa)	30.34
Tensile modulus, *E*_1_ (electrode direction) (GPa)	15.86
Poisson’s ratio, *v*_12_	0.31
Poisson’s ratio, *v*_21_	0.16
Shear modulus, *G*_12_ (GPa)	5.515
*d*_33_ (rod direction) (pC/N)	400
*d*_31_ (electrode direction) (pC/N)	−170

**Table 2:** Harvester characterization at 0.5 g excitation level, using the 2151A power management board.

Patch	*f* (Hz)	*V_out_* (V)	*I_out_* (μA)	*R* (kΩ)	*P_out_* (μW)
Outer (// connection)	65.27	2.615	125.0	21.00	653.8
Inner		1.0	0.256	21.00	0.512
Outer (// connection)	78.35	0.381	18.20	22.00	13.87
Inner		2.305	105.1	22.00	484.5

**Table 3:** Efficiency comparison of the power management boards used as conditioning circuits for the designed harvester.

Board Type	*V_in_* (V)	*I_in_* (μA)	*R* (kΩ)	*V_out_* (V)	*I_out_* (μA)	Efficiency (%)
bq25570EVM-206	3.73	134.5	13.0	1.8	137.5	49.4
2151A	4.42	106.0	14.5	1.8	124.5	47.8

**Table 4:** Parameter ranges for the design optimization.

Parameter	Reference Value (mm)	Lower Bound (mm)	Upper Bound (mm)
$L_{o}$	80	40	120
$b_{o}$	10	5.0	15.0
$L_{c}$	10	5.0	15.0
$b_{c}$	1.0	0.5	1.50
$L_{i}$	60	23	113
$b_{i}$	9.0	5.0	15.0
t ^1^	1.0	0.5	1.50

^1^ Discrete parameter since it is limited to commercial sheet metal; step size 0.5 mm.
